# Sunscreen Label Marketing Towards Pediatric Populations: Guidance for Navigating Sunscreen Choice

**DOI:** 10.7759/cureus.46785

**Published:** 2023-10-10

**Authors:** Lauren Druml, Amber M Ilyas, Erum N Ilyas

**Affiliations:** 1 Dermatology, Drexel University College of Medicine, Philadelphia, USA; 2 Health and Environmental Impact, AmberNoon, King of Prussia, USA; 3 Research & Development, AmberNoon, King of Prussia, USA

**Keywords:** sunscreen marketing, sunscreen label, spf, pediatric dermatology, sunscreen

## Abstract

Introduction: Sunscreen marketing to specific demographics is largely unregulated. Marketing specifically targeting pediatric populations has the potential to drive consumer behavior. The American Academy of Pediatrics (AAP) and American Academy of Dermatology (AAD) provide recommendations for sunscreen use in children over the age of six months. This study sought to determine if sunscreen products marketed toward pediatric populations align with healthcare guidelines.

Materials and methods: Sunscreens available in major retail outlets in the Philadelphia area were cataloged and reviewed for marketing targeting specific demographics such as “baby”, “babies”, “children”, “kids”, “sports”, and “active”. The products were reviewed for sun protection factor (SPF), broad-spectrum ultraviolet (UV) protection, water resistance, active UV filters, and application method.

Results: Of 410 sunscreens cataloged, 27 were marketed towards “baby” or “babies”, 44 towards “children” or “kids”, and 71 towards “sports” or “active”. All of the sunscreen products reviewed targeting the pediatric population offered water resistance for up to 80 minutes and broad-spectrum UV coverage. Sunscreens targeting “baby” or “babies” aligned most closely with AAP guidelines for sunscreen use in pediatric populations, with 92.6% offering an SPF between 15 to 50 and no products including oxybenzone as a UV filter. However, sunscreens targeting “children”, “kids”, “sports”, and “active” bore a close resemblance to the overall sunscreen profile for all demographics but with a higher percentage of products containing oxybenzone. Oxybenzone was found in 11.4% of “children” and “kids” products and 16.9% of “sports” and “active” sunscreen products, compared to 7.6% of all sunscreen products available, and was also found in most sunscreen products with an SPF of 70 or higher.

Conclusion: Sunscreen products marketed towards “baby” and “babies” tend to align closely with guidelines for sunscreen use in the pediatric population for children over six months of age; however, those with brand marketing towards “children”, “kids”, “sports”, and “active” do not. Limiting recommendations for a sunscreen product with an SPF of 30 to 50 targeting this demographic, however, sufficiently meets guidelines set forth by the AAP and AAD.

## Introduction

Exposure to ultraviolet (UV) radiation from the sun has been linked to a spectrum of adverse health effects, including sunburn, actinic damage, and increased susceptibility to skin cancer. [1] Children and babies are particularly at risk for cumulative UV damage, with the impact of UV damage noted in the first year of life, given that their skin is relatively immature as both a barrier and from an immunologic perspective. [2] It is thought that melanocytes in the skin of children are more susceptible to UV damage that may increase the risk of skin cancer as an adult. [3] Because of this heightened vulnerability, sun protection in this demographic is of great importance. 

For consumers seeking sunscreen, there is widespread marketing of sunscreen products targeting children and babies based on the labeling of products by brands as specifically intended for this demographic. Applying labels that include demographic marketing terms such as "baby", “kids”, or “sports” on sunscreen products is largely unregulated in the United States. [4] With guidelines for sunscreen recommendations by the AAD and AAP for children and babies and marketing by brands targeting this demographic, the question arose as to whether these efforts are aligned or if there is a gap between sunscreen marketing claims and pediatric guidelines for sunscreen use. 

This study sought to investigate sunscreen products marketed specifically for children and babies and available across various retail outlets. By reviewing these products, a better understanding of industry-driven marketing for this demographic will help educate parents, caregivers, and healthcare professionals about the ingredients of these various sunscreen products, thereby contributing to the ongoing discourse surrounding skin health and safety in these vulnerable populations.

## Materials and methods

It was determined that this study did not involve the use of human subjects, and IRB approval was not necessary. This research was structured as an observational analysis of sunscreen products marketed for children and babies. The investigation entailed a comprehensive assessment of suncare and sun protection displays within four prominent retail establishments in the Philadelphia region: CVS, Walgreens, Target, and Wegmans. The study was conducted between June 1, 2023, and June 10, 2023. A combined approach of physical in-store assessment and online reviews was utilized to ensure the inclusion of all available products. The physical locations of the selected major retail outlets were visited. Additionally, the websites of the respective sunscreen manufacturers were reviewed to capture both in-store and online product offerings, accounting for any potential regional variations in inventory.

The available sunscreens, both in-store and online, were cataloged, capturing key parameters, including (1) brand name, (2) product type (e.g., lotion, spray, stick, lip balm), (3) sun protection factor (SPF), (4) claims for broad-spectrum protection, (5) active sunscreen ingredients, (6) water resistance, and (7) specific marketing descriptors such as "kids," “children,” and "baby." 

A detailed analysis of active ingredients was conducted for all sunscreen formulations. This analysis focused on identifying ingredients capable of delivering UVB and UVA protection, which is necessary for evaluating the sun protection effectiveness of the examined products. A total of 410 distinct sunscreen varieties were cataloged and reviewed as part of this study. The collected data was then systematically organized and subjected to statistical analysis using simple descriptive statistics to discern the overall properties of sunscreen products by SPF, active ingredients, broad spectrum, water resistance, and method of application for sunscreen products formulated for babies and children. 

## Results

Out of the total 410 sunscreens cataloged, 380 (93%) offered an SPF of 30 or higher. All (100%) of the evaluated sunscreens claimed to offer broad-spectrum UV coverage. All of these products had disclaimers under the “directions” portion of the back product label stating in fine print, “children under 6 months of age: ask a doctor.” Full data is available from the authors.

**Figure 1:**
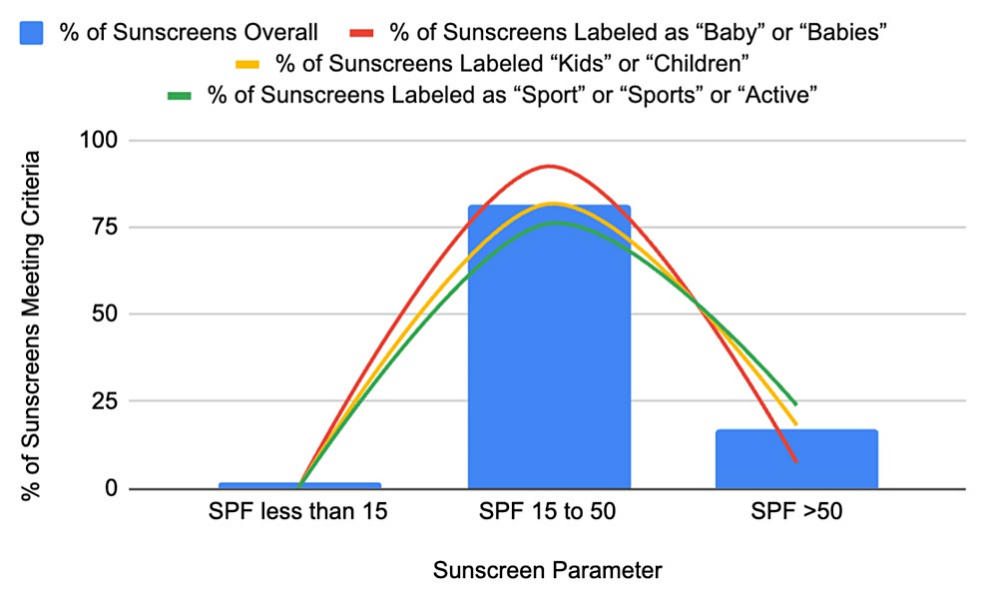
SPF distribution of sunscreen products based on pediatric demographic marketing.

The SPF ratings varied from 4 to 100. There were six (1.5%) sunscreen products that offered an SPF ranging from 4 to 12. For SPF ratings between 15 and 50, there were 335 (81.7%) products available, with 23 (5.6%) offering an SPF of 15, 1 (0.2%) an SPF of 25, 113 (27.6%) an SPF of 30, 1 (0.2%) an SPF of 35, 2 (4.9%) an SPF of 40, 13 (3.2%) an SPF of 45, and 182 (44.4%) with an SPF of 50. There were 69 (16.8%) remaining products offering an SPF >50, with six (1.5%) offering an SPF of 55, 13 (3.2%) offering an SPF of 60, five (1.2%) offering an SPF of 65, 29 (7.1%) offering an SPF of 70, 1 (0.2%) offering an SPF of 90, and 15 (3.7%) offering an SPF of 100. There were 30 sunscreen products with an SPF of less than 30 (7.3%) and 380 (92.7%) with an SPF of 30 or higher (Figure 1).

Water resistance was offered by 379 (92.4%) sunscreens, of which 371 (97.9%) had 80 minutes of water resistance, seven (1.8%) had 40 minutes of water resistance, and one (0.3%) claimed 30 minutes of water resistance. 

With regard to the method of application, of the 410 sunscreens cataloged, most products were lotions, sprays, or sticks, with 209 (51%) lotions with SPF ratings ranging from 4 to 100, 129 (31.5%) sprays with SPF ratings from 12 to 100, and 35 (8.5%) sticks with SPF ratings from 30 to 70. The remainder of the sunscreens were one (0.2%) as a cream with an SPF of 15, one (0.2%) as a hair mist with an SPF of 30, one (0.2%) powder with an SPF of 30, two (0.5%) serums with an SPF of 60, and three (0.7%) roll-ons with SPF ratings from 50 to 60. Four (1.0%) is listed as a gel with SPF ratings ranging from 30 to 50; five (1.2%) is listed as an oil with SPF ratings ranging from 4 to 50; 9 (2.2%) is listed as a lip balm with SPF ratings ranging from 15 to 50; and nine (2.2%) is listed as a mist with SPF ratings ranging from 30 to 100.

**Figure 2:**
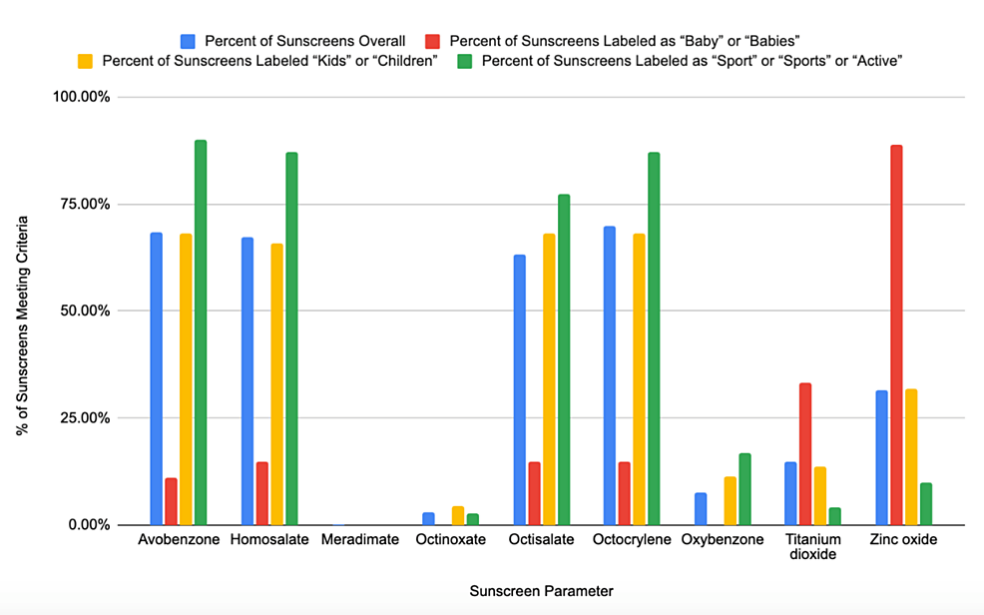
UV filters are used in sunscreen formulations based on demographics targeted at the pediatric population

Active sunscreen ingredients consisted of products containing only chemical or organic sunscreen ingredients, only physical or inorganic sunscreen ingredients, or products containing a combination of both. There were 281 out of 410 sunscreens (68.5%) containing exclusively chemical sunscreen ingredients, 117 (28.5%) with only physical sunscreen ingredients, and 12 (2.9%) containing a combination of both. Of the 293 sunscreens that had chemical sunscreen ingredients, avobenzone was found in concentrations ranging from 1% to 3% in 281 (68.5% of the total number of sunscreens), homosalate in concentrations ranging from 1% to 15% in 276 (67.3%), octisalate in concentrations ranging from 1% to 5% in 259 (63.2%), octocrylene in concentrations ranging from 1% to 10% in 286 (69.8%), oxybenzone concentrations ranging from 2% to 6% in 31 (7.6%), octinoxate in concentrations ranging from 2% to 7.5% in 12 (2.9%), and meradimate in a concentration of 5% in one (0.2%). Of the 129 (31.5%) sunscreens that included physical sunscreen ingredients, titanium dioxide in concentrations ranging from 3% to 20.8% in 61 (14.9% of the total number of sunscreens) products, and zinc oxide in concentrations ranging from 2.4% to 24.8% in all sunscreen formulations that included physical UV filters (31.5%) (Figure 2).

The subset of sunscreens explicitly marketed for children and babies was the focus of this study. Among the 410 sunscreens cataloged, 71 (17.3%) were identified as being marketed to this demographic based on the labeling that included the terms “children”, “kids”, “baby”, or “babies”. Of the 71 sunscreens marketed to this demographic, 27 were marketed toward “baby” or “babies”, and 44 were marketed toward “children” or ”kids”. Further review of the labeling on these products did not define an age for each of these demographics (Table 1).

**Table 1:** Sunscreen parameters are determined by demographic marketing based on SPF, broad spectrum, water resistance, UV filter, and method of application.

Sunscreen Parameter	Percent of Sunscreens Overall	Percent of Sunscreens With Marketing Targeting “Baby” or “Babies”	Percent of Sunscreens With Marketing Targeting “Kids” or “Children”	Percent of Sunscreens With Marketing Targeting “Sport” or “Sports” or “Active”
SPF				
SPF less than 15	1.5%	0%	0%	0%
SPF 15 to 50	81.7%	92.6%	81.8%	76.1%
SPF ≥ 30	92.7%	100%	100%	91.5%
SPF > 50	16.8%	7.4%	18.2%	23.9%
Broad Spectrum				
Claims of Broad Spectrum	100%	100%	100%	100%
Water Resistant				
80 minutes	92.4%	100%	100%	100%
UV filters				
Avobenzone	68.5%	11.1%	68.2%	90.1%
Homosalate	67.3%	14.8%	65.9%	87.3%
Meradimate	0.2%	0%	0%	0%
Octinoxate	2.9%	0%	4.5%	2.8%
Octisalate	63.2%	14.8%	68.2%	77.5%
Octocrylene	69.8%	14.8%	68.2%	87.3%
Oxybenzone	7.6%	0%	11.4%	16.9%
Titanium dioxide	14.9%	33.3%	13.6%	4.2%
Zinc oxide	31.5%	88.8%	31.8%	9.9%
Method of Application				
Lotion	51%	52%	31.8%	39.4%
Spray	31.5%	29.6%	47.7%	46.5%
Stick	8.5%	18.5%	13.6%	8.5%
Roll on	0.7%	0%	6.8%	2.8%
Lip Balm	2.2%	0%	0%	2.8%
Mist	2.2%	0%	0%	0%
Serum	0.5%	0%	0%	0%
Gel	1.0%	0%	0%	0%
Oil	1.2%	0%	0%	0%
Hair Mist	0.2%	0%	0%	0%
Cream	0.2%	0%	0%	0%
Powder	0.2%	0%	0%	0%

Baby sunscreen

Of the 71 sunscreens marketed to the pediatric demographic, 27 were marketed toward “baby” or “babies”. Further review of the labeling on these products did not define a specific age for this demographic. Consistent with the directions listed on all sunscreen products cataloged, on the back label under directions, a disclaimer stating “children under 6 months of age: ask a doctor” was present on all “baby” and “babies” products reviewed.

Of the 27 sunscreen products targeting babies, 25 (92.6%) featured an SPF of between 15 and 50, while two products had an SPF of 55. There were no products designated for infants with an SPF over 55. All (100%) of these sunscreen products had an SPF greater than 30 (Figure 1).

Of the 27 “baby” or “babies” sunscreen products, 23 (85%) contained only physical UV filters, while three (11%) contained chemical UV filters, and one (3.7%) had a combination of both physical and chemical UV filters. The physical UV filters included zinc oxide and titanium dioxide. Fourteen (52%) contained zinc oxide as the exclusive UV filter, with concentrations ranging from 12% to 24.8%. There were nine (33%) baby sunscreen products that combined physical UV filters with zinc oxide and titanium dioxide. All three baby sunscreen products containing chemical UV filters without physical filters included avobenzone, homosalate, octisalate, and octocrylene as active ingredients. The final product was labeled “mineral enriched” and contained three chemical UV filters, homosalate, octisalate, and octocrylene, along with one mineral UV filter, zinc oxide. 

Of the four sunscreens that contained chemical sunscreen ingredients, all four contained homosalate at a concentration of 9% and octisalate at a concentration ranging from 3% to 4.5%. Avobenzone in concentrations ranging from 2.7% to 3% and octocrylene in a concentration of 9% were found in three (11.1%) of chemical sunscreens containing baby products. Oxybenzone and meradimate were not found in any baby sunscreen product reviewed (Figure 2).

Of the 24 sunscreens that included physical sunscreen ingredients, titanium dioxide in concentrations ranging from 3.4% to 8.1% was seen in nine (33.3%) products. All baby sunscreen products that included physical sunscreen ingredients contained zinc oxide in concentrations ranging from 7.3% to 24.8%.

All of the sunscreen products with “baby” or “babies” on the label were found to be water-resistant for up to 80 minutes and have a broad spectrum. The method of application for 14 of the 27 (52%) baby sunscreens were lotions, five (18.5%) were sticks, and eight (29.6%) were spray formulations.

Children's sunscreen

Of the 71 sunscreens marketed to the pediatric demographic, 44 were marketed to “children” or ”kids”, with no precise definition offered to define the age group to define this demographic. Consistent with the directions listed on all sunscreen products cataloged, on the back label under directions a disclaimer stating “children under 6 months of age: ask a doctor” was present on all “children” and “kids” products reviewed.

Of the 44 sunscreen products targeting children, 36 (81.8%) offered an SPF between 15 and 50, with no products offering an SPF below 15. There were eight (18.2%) sunscreen products offering an SPF greater than 50, with three (6.8%) offering an SPF of 100. All sunscreen products in this category had an SPF of 30 or higher (Figure 1).

As for active ingredients in children's sunscreen products, 13 (29.5%) contained only physical UV filters, while 31 (70.5%) contained chemical UV filters, and one (2.3%) had a combination of both physical and chemical UV filters. The physical UV filters included zinc oxide and titanium dioxide. Seven (15.9%) contained zinc oxide as the exclusive UV filter, with concentrations ranging from 15% to 24.8%. There were six (13.6%) children's sunscreen products that combined physical UV filters, zinc oxide, and titanium dioxide. For the product combining physical and chemical filters, zinc oxide was combined with the chemical UV filters octinoxate and octisalate (Figure 2).

Of the 31 children's sunscreen products without physical UV filters, the chemical sunscreens included homosalate, octinoxate, octisalate, octocrylene, and oxybenzone as potential active ingredients. Of these 31 sunscreens, avobenzone in concentrations ranging from 2.5% to 3% was found in 30, homosalate in a concentration of 9% to 15% in 29 products, octisalate in a concentration ranging from 4.5% to 5% in 30, octocrylene in a concentration of 4% to 10% in 30, oxybenzone in concentrations ranging from 3.5% to 6% in five sunscreen products, and octinoxate in concentrations ranging from 3% to 7.5% in two. None of the children’s sunscreen products were found to contain meradimate. Of note, five of the six sunscreen products with an SPF of 70 or higher contained oxybenzone, and all three sunscreen products with an SPF of 100 contained oxybenzone.

All of the sunscreen products with “children” or “kids” on the label were found to be water-resistant for up to 80 minutes and have a broad spectrum. The method of application for 14 of the 44 (31.8%) children's sunscreens were lotions, 21 (47.7%) were spray formulations, six (13.6%) were sticks, and three (6.8%) were roll-ons.

Sports formulations

Concurrently, we examined 71 sunscreens designated as "active", "sport", or “sports”, which are categories that may be geared toward children engaged in sporting activities. Examination of the label did not reveal details to specify age or define the type of activity, although similar to all products cataloged, a standard disclaimer on the back label under directions stating “children under six months of age: ask a doctor” was present on all products reviewed.

Of the 71 sunscreen products targeting “sport”, “sports”, and “active”, 54 (76.1%) offered an SPF of between 15 and 50, with no products offering an SPF below 15. There were 17 (23.9%) sunscreen products offering an SPF greater than 50, with five (7.0%) offering an SPF of 100. Of these sunscreen products, six (8.5%) had an SPF of less than 30, while 65 (91.5%) had an SPF of 30 or higher.

For sports and active-related sunscreens, seven (9.9%) formulations contained physical UV filters, while 64 (90.1%) contained chemical UV filters. There were no products that combined physical and chemical UV filters. The physical UV filters included zinc oxide and titanium dioxide, with all seven containing zinc oxide in concentrations ranging from 6.5% to 24.08% and three combined with titanium dioxide in concentrations ranging from 3.4% to 4.5%. 

Chemical UV filters included homosalate, octinoxate, octisalate, octocrylene, and oxybenzone as potential active ingredients. Of these 64 sunscreens, avobenzone in concentrations ranging from 1.5% to 3% was found in all 64 (90.1% of sports-related sunscreens), homosalate in a concentration of 3% to 15% in 62 products (87.3%), octisalate in a concentration ranging from 4% to 5% in 55 (77.5%), octocrylene in a concentration of 4% to 10% in 62 (87.3%), oxybenzone in concentrations ranging from 3.5% to 6% in 12 (16.9%), and octinoxate in concentrations ranging from 2% to 5% in two (2.8%). None of the sport-related sunscreen products were found to contain meradimate. Of note, eight of the 11 sunscreen products with an SPF of 70 or higher contained oxybenzone, and all sunscreen products with an SPF of 100 contained oxybenzone (Figure 2).

All of the sunscreen products with “sport”, “sports”, or “active on the label were found to be water-resistant for up to 80 minutes and have a broad spectrum. The method of application for two of the 71 (2.8%) were lip balms, 28 (39.4%) were lotions, two (2.8%) were roll-ons, 33 (46.5%) were spray formulations, and six (8.5%) were sticks.

## Discussion

As parents and caregivers have become more attuned to the significance of sun protection, applying sunscreen products is one of the methods commonly recommended by healthcare professionals to achieve photoprotection for children over the age of six months. [5] The Food and Drug Administration (FDA) and the American Academy of Pediatrics (AAP) do not recommend sunscreen under the age of six months, given the risk of sensitivity to sunscreen ingredients, while noting that if sunscreen sensitivities are seen in babies over the age of six months, choose a sunscreen with zinc oxide or titanium dioxide [6,7]. The AAP guidelines for sunscreen use include recommendations for seeking “broad spectrum” sunscreens with a “sun protection factor (SPF) of at least 15 (up to SPF 50).” These guidelines further state that “an SPF of 15 or 30 should be fine for most people” with “more research studies …needed to test if sunscreen with more than SPF 50 offers any extra protection”. In terms of ingredients, the AAP guidelines specify to “avoid the sunscreen ingredient oxybenzone because of concerns about mild hormonal properties” if possible, while noting that any sunscreen is better than no sunscreen to prevent sunburn. And recommendations for zinc oxide or titanium dioxide were made for “sensitive areas of the body, such as the nose, cheeks, tops of the ears, and shoulders” [7].

The American Academy of Dermatology (AAD) recommendations for sunscreen use do not appear to differentiate between adults and children over six months of age. Consistent with the FDA and AAP recommendations, the AAD recommends avoiding sunscreen for infants under six months of age when possible. For those over six months of age, the use of broad-spectrum, water-resistant sunscreen with an SPF over 30 is encouraged [8].

The active ingredients in sunscreens, known as ultraviolet (UV) filters, are the foundation of sunscreen products as they shield the skin from harmful UV radiation. There are several UV filters, each with specific wavelengths of UV light filtered. Given the varying profiles for each UV filter, different combinations of filters may be combined to achieve UVB blockage for the sun protection factor (SPF) listed, in addition to potential UVA blockage to achieve broad spectrum coverage [9]. 

The UV filters used in sunscreen products tend to fall under chemical or organic UV filters and physical or inorganic filters. In this study, the chemical sunscreens found in the products reviewed included avobenzone, homosalate, meradimate, octocrylene, octinoxate, oxybenzone, and octisalate. The most common chemical sunscreens found in sunscreens marketed to pediatric populations were avobenzone, homosalate, octisalate, and octocrylene. For sunscreens marketed towards “children” and “kids”, the distribution of these ingredients was similar as compared to the general profile of sunscreens available and substantially higher for those marketed towards “sports” and “active”. For sunscreens marketed towards “baby” and “babies”, chemical sunscreen ingredients were far less frequently used. Sunscreens marketed to pediatric populations did not contain meradimate. Octinoxate was not found in sunscreens marketed to “baby” and “babies” and was more often found in “children” and “kids” sunscreens compared to the general sunscreen profile or those marketed to “sports” and “active”. Oxybenzone was not found in “baby” or “babies” sunscreens; however, it was found more often in “children” and “kids” sunscreens than in the general profile of sunscreens available and found in more than twice the number of sunscreens marketed to “sports” and “active”. Oxybenzone use in sunscreen products has gained attention in recent years due to its possible impact on the environment [10], potential estrogenic effects [11], possible association with Hirschsprung’s disease [12], and systemic absorption [13]. Further research is needed to understand the impact on health and the environment. Although the AAP comments on avoiding oxybenzone when possible, the AAD recommendations for sunscreen use do not at this time [7,8]. For the products reviewed in this study, oxybenzone was only found in sunscreen products with an SPF of 70 or higher.

The physical UV filters found in the sunscreen products reviewed included titanium dioxide and zinc oxide. A study by Matta et al. demonstrated that all avobenzone, homosalate, octisalate, octinoxate, octocrylene, and oxybenzone are systemically absorbed with plasma concentrations that exceeded FDA thresholds [13]. Given the unclear impact on health, more studies are warranted. The general guidance for patients who express concerns about this information is to seek sunscreens that use physical UV filters such as zinc oxide or titanium dioxide [14]. Sunscreens marketed to “baby” and “babies” were found to contain zinc oxide in the majority of products (88.8%) and titanium dioxide in one-third of products (33.3%). For those products marketed to “children” and “kids”, however, the distribution of titanium dioxide and zinc oxide was similar to the general profile of sunscreens. Only 4.2% of sunscreens marketed towards “sports” or “active” contained titanium dioxide, and only 9.9% contained zinc oxide (Figure 2).

Marketing towards a pediatric demographic of consumers that includes baby/babies, kids/children, and sports/active labeling may appeal to parents seeking options tailored to their child’s particular skincare needs. With growing awareness of the need for photoprotection for children, sun care brands may tend to target this consumer base. Concerns about sunscreen labeling and consumer comprehension of sunscreen terminology are well-founded. A study conducted by Shiosaki et al. investigated the sources of information that may influence parents’ opinions on sunscreen. They found that online information about the benefits and risks of sunscreen use was variable and incomplete [15]. A study by Kong et al. evaluating influences on consumer choice for particular sunscreen products tended to focus primarily on the SPF value, sensitive skin marketing, and water resistance [16]. Tribby et al. demonstrated that SPF was the primary determinant driving consumer choice for sunscreen, not active sunscreen ingredients [17]. While Prado et al. demonstrated that online purchases for sunscreen products tend to be driven primarily by the number of reviews and unregulated marketing claims [18].

In reviewing sunscreen labels for the purposes of this study, specific age definitions to fit each demographic were not available to determine age cutoffs for babies, children, and those engaged in sports. The AAP also only distinguishes between infants less than six months of age and those above six months of age in defining sunscreen guidelines. The AAP and the FDA specify that infants under the age of six months should avoid direct sunlight and not apply sunscreen [6,7]. Unfortunately, labeling for sunscreen products does not tend to state this in the marketing terminology or on the front of a sunscreen label. This information is buried under “directions” on the back of the label, where it stands to reason many consumers may not read. Without clear age definitions, parents may mistakenly assume the term “baby” or “babies” to apply to any child under the age of one without excluding those under the age of six months. 

The results of this study determined that sunscreens with “baby” or “babies'' in the marketing were aligned most closely with AAP guidelines for sunscreen use in children over the age of six months, with 92.6% of sunscreen products falling between an SPF of 15 and 50 and no products containing oxybenzone. Sunscreen products marketed towards children and kids, on the other hand, bore a much closer resemblance to the general profile for sunscreen products, with 81.8% having an SPF of 15 to 50 compared to 81.7% overall. However, 11.4% of sunscreen products targeted at children through labeling contain oxybenzone, compared to the overall sunscreen profile of 7.6% containing oxybenzone. Sunscreens targeting sports and active demographics tended to deviate from AAP guidelines, with only 76.1% of these products having an SPF of 15 to 50, 23.9% having an SPF rating over 50 up to 100, and 16.9% containing oxybenzone.

Based on AAD recommendations, all of the sunscreen products marketed to “baby”, “babies”, “children”, and “kids” met the criteria for broad spectrum, water resistance, and having an SPF of 30 or higher. For sunscreens marketed as “sports” or “active”, only 91.5% of sunscreens meet the SPF criterion. This was even less than the number of sunscreens meeting this criterion based on the overall profile of the SPF of all sunscreen products evaluated. The AAD does not include recommendations on sunscreen filters to avoid [8].

With SPF shown as a driving factor for consumer sunscreen purchase, finding an SPF with pediatric targeted marketing that fits healthcare guidelines provided by the AAP and AAD can help consumers find a sunscreen best suited for their child’s sun protection needs. Sunscreens targeting “babies”, “baby”, “children”, and “kids” meet AAD recommendations. For consumers seeking sunscreens marketed for “sports” or “active”, choosing a product with at least an SPF of 30 is reasonable, given that all sunscreen products targeting this demographic meet the criteria for broad spectrum and water resistance. For parents seeking to meet AAP guidelines, choosing a sunscreen product with an SPF of 15 to 50 with demographic marketing directed towards “baby”, “babies”, “children”, “kids”, “sports”, or “active” would be sufficient. Meeting AAP guidelines would include avoiding the use of oxybenzone in sunscreen products. Oxybenzone was only present in sunscreen products with an SPF of 70 or higher. Sunscreen products with an SPF of 15 to 50 did not contain oxybenzone. To fit the criteria offered by both the AAD and AAP for sunscreen use in children, choosing a sunscreen product targeting pediatric demographics with an SPF of 30 to 50 would be sufficient. 

There are several limitations to this study. The first limitation was that we did not examine the inactive ingredients found in sunscreen products. Tribby et al. demonstrated that consumers were more likely to read ingredient labels to determine products to avoid based on the presence of ingredients they intended to avoid, as opposed to choosing products based on recommended active ingredients. [17] Phadungsaksawasdi et al. determined that sunscreens marketed to pediatric consumers contain common allergens, and Kong et al. demonstrated that marketing toward sensitive skin influenced consumer choice of sunscreen products [16,19]. The presence of contact allergens in products targeting pediatric consumers may increase the risk of skin rashes; however, the AAP does not include guidelines on specific inactive ingredients to avoid. The AAD recommends choosing zinc oxide or titanium dioxide for those with sensitivities but does not offer guidance on specific ingredients to avoid [8]. The second limitation is that this study was limited to brands purchased at retail outlets. With increased online shopping and the study by Prado et al. demonstrating consumer behavior online as driven by product reviews, there is a risk of further unregulated claims found in individual reviews that may drive consumer behavior. [18] Tamminga et al. found higher levels of engagement with misinformation on sunscreens presented on parenting blogs that may also serve to drive consumer sunscreen choice in this population. [20] However, reviewing in-store options followed by website reviews for each brand was intended to be as inclusive as the sunscreen product variants available. And lastly, this study did not take into consideration products marketed specifically towards sensitive skin; however, marketing towards sensitive skin tends to encompass a broader demographic of consumers that are of both pediatric and adult age groups. Despite these limitations, it is clear that marketing toward pediatric populations does not encompass healthcare guidelines for sunscreen products in this vulnerable population.

## Conclusions

Sunscreen products marketed towards “baby” and “babies” tend to align closely with guidelines for sunscreen use in the pediatric population for children over six months of age. However, this study demonstrated that sunscreen products marketed towards children and those engaged in sports showed a remarkable similarity in active ingredient compositions to the overall profile of regular sunscreens available on the market without taking into consideration healthcare societies' guidelines for sunscreen products in the pediatric population. However, with SPF as a primary determinant for sunscreen choice amongst consumers, the findings of this study suggest that recommending sunscreen products targeting pediatric demographics with an SPF of 30 to 50 would sufficiently meet the guidelines offered by both the AAD and AAP for sunscreen products in children.
